# Action Shapes the Sense of Body Ownership Across Human Development

**DOI:** 10.3389/fpsyg.2018.02507

**Published:** 2018-12-17

**Authors:** Elena Nava, Chiara Gamberini, Agnese Berardis, Nadia Bolognini

**Affiliations:** ^1^Department of Psychology and NeuroMi – Milan Center for Neuroscience, University of Milano–Bicocca, Milan, Italy; ^2^Laboratory of Neuropsychology, IRCSS Istituto Auxologico Italiano, Milan, Italy

**Keywords:** body ownership, development, motor system, sensorimotor integration, rubber hand illusion

## Abstract

In this study we investigated, both in childhood and adulthood, the role of action in promoting and shaping the sense of body ownership, which is traditionally viewed as dependent on multisensory integration. By means of a novel action-based version of the rubber hand illusion (RHI), in which participants could actively self-stroke the rubber hand, with (Version 1) or without visual feedback (Version 2) of their own actions, we showed that self-generated actions promote the emergence of a sense of ownership over the rubber hand in children, while it interferes with the embodiment of the rubber hand in adults. When the movement is missing (Version 3, i.e., mere view of the rubber hand being stroked concurrently with one’s own hand), the pattern of results is reversed, with adults showing embodiment of the rubber hand, but children lacking to do so. Our novel findings reveal a dynamic and plastic contribution of the motor system to the emergence of a coherent bodily self, suggesting that the development of the sense of body ownership is shaped by motor experience, rather than being purely sensory.

## Introduction

The sense of body ownership is the product of complex mechanisms, primarily relying on the binding of multisensory body-related signals. Studies investigating the neural principles of the bodily-self have shown that multisensory inputs converge into a fronto-parietal network, in which they likely contribute to the building of a unique, sensory-based percept of the sense of body ownership (see Blanke et al., 2015 for a recent review). Interestingly, studies in adult animals and humans have shown that a crucial role in this network is played by the premotor cortex (Graziano, 1999; Ehrsson et al., 2004), which mediates sophisticated sensorimotor interactions relevant for action and the representation of the whole body and its single parts in space.

Some aspects of body representation may be innate, as suggested by studies conducted in patients with congenital limb aplasia, a condition in which individuals were born with one or more missing limbs (Melzack et al., 1997; Brugger et al., 2000). Despite complete absence of the physical limb, and thus the experience of seeing, touching, and moving it, a number of patients report phantom limb sensations, suggesting that the neural representation of the body may be partially genetically programmed.

In support to the claim that there may be a predisposition to some aspects of body representation, studies conducted in human newborns have revealed that within the first hours of life, newborns look longer to an image of a baby face being stroked concurrently with one owns face than an image of a baby face being stroked asynchronously. Interestingly, this preference is abolished in both synchronous and asynchronous stroking mode when the face is inverted by 180°, suggesting that newborns have a rudimentary sense of self (Filippetti et al., 2013, 2015).

An adult-like sense of body ownership seems to gradually develop in humans, and mostly depend upon multisensory integration skills. These skills have a protracted development in childhood, in that they are suboptimal until at least 8 years of age (Gori et al., 2008; Gori, 2015). Before this age, children are mostly dominated by one sensory modality at a time, which likely calibrates the others. Evidence that the sense of body ownership depends upon multisensory integration skills come from recent studies investigating the sense of body ownership in preschool children, showing that children are insensitive to classical multisensory bodily illusion, such as the RHI. Absence of recalibration of own hand’s position toward the rubber hand has typically been interpreted as children’s inability to fuse the multisensory information necessary to embody the rubber hand (Cowie et al., 2013, 2016; Nava et al., 2017).

So far, studies using the RHI to assess body ownership in adults and children have focused on the underlying multisensory mechanisms (Serino et al., 2013; Blanke et al., 2015), largely neglecting the potential existence of a motor side of this component of the bodily self. However, neuroimaging and non-invasive brain stimulation studies have revealed that activity in the premotor cortex (Ehrsson et al., 2004, 2005; Convento et al., 2018) is associated with feelings of owning the rubber hand, and that neurons in the ventral premotor cortex react to multisensory stimuli that guide action (Graziano et al., 1994; Fogassi et al., 1996). That is, motor functions are strictly interconnected to sensory feedback and are at the roots of the body schema.

Furthermore, there is a growing body of evidence in healthy and brain-damaged adult patients, indicating that the motor system may shape and guide the emergence of a multisensory bodily self, in general, and of the sense of body ownership, in particular (Vallar and Ronchi, 2009; Garbarini et al., 2013; Bolognini et al., 2014; Hara et al., 2015; della Gatta et al., 2016). For instance, in adults, della Gatta et al. (2016) showed that the illusory ownership of a rubber hand, brought about by the RHI, is accompanied by a decrease of motor cortical excitability in the participants’ real disembodied hand, as measured through motor evoked potentials induced by transcranial magnetic stimulation (TMS) of the primary motor cortex (M1). Accordingly, down-regulating the excitability of M1 by means of repetitive TMS attenuates the sense of body ownership, in turn rendering individuals more prone to incorporate an alien body through the RHI (Fossataro et al., 2018). Even on a more extreme hand, individuals whose limb was immobilized for 1 week show stronger RHI effects on the immobilized hand (Burin et al., 2017) after this period, suggesting that being able to performed self-generated movements has a crucial role in shaping the experience of one’s own body.

The strong link between movements and body ownership is also well proved by neuropsychological evidence: patients with upper-limb hemiplegia following an acquired stroke are more susceptible to the RHI (Burin et al., 2015; see also Nava et al., 2017), further suggesting that impairment of the motor system directly affects the multisensory sense of body ownership. Furthermore, in Critchley’s (1953) taxonomy, seminal in the neuropsychological literature, somatoparaphrenia, namely a delusion of disownership of contralesional body parts seldom observed after a stroke (Vallar and Ronchi, 2009), is closely associated with unawareness and active denial of motor deficits.

While the relationship between the motor and sensory systems has received attention in healthy adult and neuropsychological studies, to date no study has investigated the role of the motor system in the construction of a coherent sense of body ownership during development.

In this framework, the present study explores whether and how self-produced actions may shape body ownership across human development, by testing both children and adults on a novel, action-based version of the RHI, in which participants could actively stroke the rubber hand (Version 1 and Version 2), as compared to the standard, purely sensory, version of the RHI (Version 3) (see Botvinick and Cohen, 1998).

Our manipulation may sound very similar to the somatic RHI introduced by Ehrsson et al. (2005), in which blindfolded participants touch the rubber hand while the experimenter touches the participant’s hand concurrently, leading to the sensation of owning the rubber hand. However, our action-based version of the RHI differs from the somatic RHI in that here participants have to actively stroke the rubber hand with a brush, always watching the rubber hand (both Versions 1 and 2). On the contrary, in Ehrsson et al. (2005) the participants’ hand was passively moved from the experimenter over the rubber hand. This is an important difference, because the aim of the study was precisely to assess the role of self-generated movements on the sense of embodiment.

## Materials and Methods

### Participants

One hundred and eight children and 108 adults took part in the experiment, and were assigned to one of the three versions of the experiment as follows: 36 children (mean age = 5.0, *SD* = 0.7, 18 females) and 36 adults (mean age = 25.1, *SD* = 4.5, 22 females) took part in Version 1; 36 children (mean age = 4.6, *SD* = 0.5, 15 females) and 36 adults (mean age = 24.5, *SD* = 4.4, 23 females) took part in Version 2; 36 children (mean age = 5.0, *SD* = 0.7, 18 females) and 36 adults (mean age = 26.6, *SD* = 7.2, 22 females) took part in Version 3. In every version of the experiment, the sample slightly exceeded the computed required sample size (*N* = 64), as calculated with G Power, with an expected Effect size = 0.25, α = 0.05, Power = 0.90.

Ten additional children (across versions) were tested but excluded from the final sample because they either did not want to continue the experiment (*N* = 5) or did not understand the task (*N* = 5).

All children were recruited from local kindergartens. All were cognitively and neurologically healthy and took part in the experiment after at least one parent gave written informed consent to participating in the study.

Adults were recruited from the University of Milan-Bicocca, received course credits for their participation, and signed and informed consent prior to the beginning of the experiment. All adult participants were right-handed by self-report. For children, we asked them to write their name on a sheet of paper, and to tell which hand they use to, e.g., brush teeth, hold a spoon.

In every version of the experiment, children and adults were split into two groups, with 50% assigned to one of the two testing conditions, corresponding to type of stroking - synchronous or asynchronous; that is, in each version the overall groups were 4 (2 for each age and type of stroking). This between-subjects design was aimed at minimizing testing time, particularly to make it more likely to children to stay focused throughout the testing.

More importantly, while the use of a between-subjects design may appear less robust than using a within-subjects design, it should be noted that re-testing the same individual on different conditions (e.g., synchronous and asynchronous) causes carry-over effects on the proprioceptive drift. For example, data from 30 adults (see Convento et al., 2018) have shown that carry-over effect persisted in these individuals even after 1 week (in which we they were not administered any test). For this reason, we opted for a between-subjects design, as previously done in other studies too (see Nava et al., 2014, 2018).

The study was approved by the Ethics Committee of the University of Milan-Bicocca, in line with the ethical principles of the Declaration of Helsinki.

### Material

A wooden horizontal surface (60 length × 40 width cm), with a vertical wooden surface (40 width × 60 height cm) attached in the middle of it served as main testing space. The participant was seated in front of the horizontal surface, with the left hand positioned behind the vertical surface, so to impede visibility of own hand. A life-sized rubber left hand was placed in front of the participant, keeping a distance of approximately 20 cm between the index finger of the hand of the participant and the index finger of the rubber hand.

**FIGURE 1:**
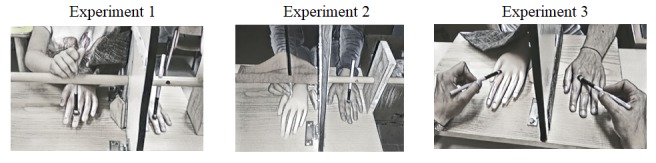
Illustrations of the three versions of the experiment, in which participants (children and adults) watch the rubber hand while actively stroking the rubber hand (Version 1), actively stroke the rubber hand with no visual feedback of the own movements (Version 2), or observe the rubber hand being stroked by another person (Version 3).

At approximately 20 cm of the vertical surface was placed a wooden rod, which was of the same length of the horizontal surface. In the rod itself, three holes were made to insert two paintbrushes at a time: in the synchronous condition, one was placed above the rubber hand, and the other above the participants’ left hand; in the asynchronous condition, one paintbrush was placed above the rubber hand, and the other 2 cm away from the participant’s left hand (see Figure 1, left panel, for a graphic description of the set-up).

In Version 2 of the experiment, the material was the same as the one used in Version 1, with the only exception of the presence of a black bloth, which was placed over the participants’ hand to prevent sight of own movements (see Figure 1, central panel).

The material used in Version 3 of the experiment resembled Version 1 and 2, with the following important difference: because the participants did not have to actively stroke the rubber hand, but passively watched the experimenter stroking the rubber hand either synchronously or asynchronously with the real hand of the participant (see Figure 1, right panel), the wooden rod was removed from the vertical surface.

### Assessment of the RHI

#### Questionnaire: Subjective Report of Body Ownership

To assess whether participants explicitly felt embodiment of the rubber hand, we administered a questionnaire composed of two simple items, similar to the ones previously used in children (Cowie et al., 2013; Nava et al., 2017). One question was designed to reflect the strength of the embodiment of the rubber hand: “I felt as if the rubber hand was my own hand,” and the other question served to control for suggestibility: “I felt as if I had three hands.” The questions corresponded to 7 possible ratings, ranging from -3 (“I strongly disagree”) to +3 (“I strongly agree”). Zero indicated “I neither agree or disagree.” For children, each rating corresponded to: +3 (“Definitely yes”), +2 (“Yes”), +1 (“A little bit), 0 (“Not sure”), -1 (“Not really), -2 (“No”), -3 (“Absolutely not”). The questions and the rating scale were read out loud, and they were repeated more times if necessary to make sure children understood the questions and the options for responding. Note that this procedure was previously used in other experiments (Cowie et al., 2013, 2016; Nava et al., 2017) in similar set-ups and the children did not manifest any problem understanding the questions and how to respond to them. Furthermore, as in Nava et al. (2017), we made use of a control question specifically to prevent from obtaining responses that could only reflect compliance with the experimenter or susceptibility to any illusion.

#### Proprioceptive Drift: Implicit Index of Self-Location in the RHI

To assess whether participants’ sense of self-location changed following induction of the illusion, we measured the pointing error, namely the so-called ‘proprioceptive drift’. This was achieved by putting the participants’ hand under a small transparent plastic table (ca 60 cm length × 40 cm height), on which a measuring tape was placed. The left middle finger was placed under the “zero” signaled by the measuring tape. Participants were required to point three times toward their left middle finger before (P1) and after the illusion induction (P2), and the difference between P2 and P1 represented the pointing error. This measure has been widely used as a behavioral, implicit index of the integration of vision, proprioception and touch, which are a necessary component of the body schema and of the sense of body ownership (Tsakiris, 2010; Convento et al., 2018).

#### General Procedure

In all 3 versions, half of the participant in each group (adults and children) were assigned to the synchronous condition, that is, the movements performed on the rubber hand were perfectly matched to the strokes provided on the participant’s hand. The other half of the participants were assigned to the asynchronous condition, in which the strokes given on the rubber hand and the real hand were not matched, thus the participant always perceived one stroke at a time, one on her own hand, followed by a stroke on the rubber hand.

In Version 1 and 2, the participants were asked to hold the paintbrush and move it along the rubber hand.

The participants were free to change the velocity during stroking.

In Version 3, the participants watched the rubber hand being stroked by the experimenter, while concurrently being stroked on owns hand.

The whole induction session for the three versions lasted ca. 3 min, with short breaks allowed every minute.

Each version of the experiment started by asking participants to make the pointing task. Immediately after, the induction of the illusion started. At the end of this phase, each participant was asked to perform the pointing task again. At last, the questionnaire was administered.

## Results

Parametric statistics was applied to both questionnaire and proprioceptive drift analyses because data were normally distributed across groups (as assessed through Shapiro-Wilks tests) and because the data were continuous.

### Subjective Report of Body Ownership

Raw scores of the two items were compared using a repeated-measures analysis of variance (rmANOVA), with Question (illusion vs. control question) as within-subjects factor, and the between-subjects factors: Group (children vs. adults), Synchrony (synchronous vs. asynchronous stroking), and Version (the 3 versions of the experiment). Newman-Keuls *post hoc* comparisons were used to explore significant interactions.

The Group X Question X Synchrony X Version rmANOVA revealed main effects of Question [*F*(1,204) = 118.74, *p* < 0.001, η^2^ = 0.37], Group [*F*(1,204) = 9.81, *p* = 0.002, η^2^ = 0.05] and Synchrony [*F*(1,204) = 36.74, *p* < 0.001, η^2^ = 0.15], and the following interactions: Version X Synchrony *F*(2,204) = 8.31, *p* < 0.001, η^2^ = 0.07], Question X Synchrony *F*(1,204) = 64.62, *p* < 0.001, η^2^ = 0.24], Group X Question X Version [*F*(2,204) = 3.86, *p* = 0.02, η^2^ = 0.04], Group X Question X Synchrony [*F*(1,204) = 8.51, *p* = 0.04, η^2^ = 0.04]. Crucially, even the Group X Question X Synchrony X Version [*F*(2,204) = 7.72, *p* = 0.001, η^2^ = 0.07] reached significance; this interaction was explored by conducting further analyses separately for the two questionnaire’s items: the illusion and the control questions.

**FIGURE 2:**
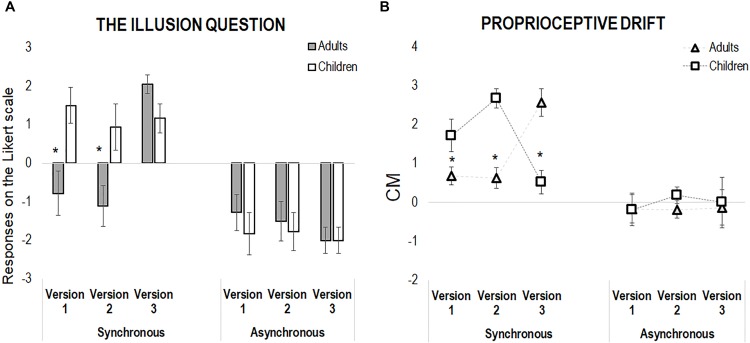
**(A)** Shows the results from the illusion question only (“Did you feel as if the rubber hand was your hand?”) in both adults and children across the three versions of the experiment, in the synchronous and asynchronous condition. **(B)** Shows the results from the proprioceptive drift, calculated as the difference between post-illusion and pre-illusion pointing, in both children and adults in the three versions of the experiment, in the synchronous and asynchronous condition. In both panels, error bars indicate standard error of the mean. The asterisks highlight differences between adults and children.

With respect to the illusion question, we found a main effect of Synchrony [*F*(1,204) = 75.31, *p* < 0.001, η^2^ = 0.27], and the following interactions: Group X Synchrony [*F*(1,204) = 6.87, *p* = 0.009, η^2^ = 0.03], Version X Synchrony [*F*(2,204) = 5.42, *p* = 0.005, η^2^ = 0.05], and Group X Version X Synchrony [*F*(2,204) = 4.59, *p* = 0.01, η^2^ = 0.04]. However, while for the asynchronous condition no main effect or interaction emerged (all *p* > 0.46), for the synchronous condition we found significant effects of Group [*F*(1,102) = 8.45, *p* = 0.004, η^2^ = 0.08], Version [*F*(2,102) = 6.60, *p* = 0.002, η^2^ = 0.12], as well as of the Group X Version interaction [*F*(2,102) = 6.68, *p* = 0.002, η^2^ = 0.12], the last revealing that children reported higher sense of body ownership in comparison to the adults in the two motor versions of the RHI, hence both in Version 1 (children: Mean, *M* = 1.50, Standard Error, *SE* = 0.48; adults: *M* = -0.78, *SE* = 0.48, *p* = 0.007) and Version 2 (children: *M* = 0.94, *SE* = 0.48; adults: *M* = -1.11, *SE* = 0.48, *p* = 0.009). On the contrary, children and adult reported comparable sense of body ownership in Version 3, when the movement was absent (children: *M* = 1.17, *SE* = 0.48; adults: *M* = 2.06, *SE* = 0.48, *p* = 0.40, see Figure 2). Morevore, within the children group, there was no difference between the three RHI versions (all *p* > 0.7), while adults did report a feeling of ownership over the rubber hand only when the action was precluded (positive score, Version 3), as compared to scores obtained in either Version 1 and 2 (all *p* < 0.001).

With respect to the control question, there was a main effect of Group [*F*(1,204) = 12.24, *p* = 0.001, η^2^ = 0.06], caused by adults rejecting the control question more than children regardless of the RHI version (*p* = and synchrony (adults: *M* = -2.60, *SE* = 0.14; children: *M* = -1.89, *SE* = 0.14), and a Synchrony X Version interaction [*F*(2,204) = 5.02, *p* = 0.007, η^2^ = 0.05], since both children and adults rejected the control question in Version 1 more than Version 3.

### Proprioceptive Drift

Recalibration toward the rubber hand was calculated as the difference between the mean of the 3 pointings performed after and before the induction of the illusion; this index represents the so-called ‘proprioceptive drift’. The proprioceptive drift was then analyzed via an univariate ANOVA, with Group (children vs. adults), Synchrony (synchronous vs. synchronous stroking), and Version (the 3 versions of the experiment) as between-subjects factors. Newman-Keuls *post hoc* comparisons were used to explore significant interactions.

This analysis revealed a main effect of Synchrony [*F*(1,204) = 53.35, *p* < 0.001, η^2^ = 0.21] and significant Group X Version [*F*(1,204) = 9.09, *p* < 0.001, η^2^ = 0.08], and Group X Version X Synchrony interactions [*F*(2,204) = 7.96, *p* < 0.001, η^2^ = 0.07].

As for the questionnaire, we conducted separate analyses for the synchronous and asynchronous conditions. While the asynchronous condition did not show any significant effect of the main factors and of their interactions (all *p* > 0.63), the analysis of the synchronous condition revealed only a significant Group X Version interaction [*F*(2,102) = 23.38, *p* < 0.001, η^2^ = 0.31]. Indeed, in both Version 1 (children: *M* = 1.72, *SE* = 0.31; adults: *M* = 0.68, *SE* = 0.31, *p* = 0.02) and Version 2 (children: *M* = 2.68, *SE* = 0.31; adults: *M* = 0.63, *SE* = 0.31, *p* < 0.001) children showed a larger proprioceptive drift in comparison to adults. Interestingly, the pattern reversed in Version 3: when no movement occurred, adults presented with a larger proprioceptive drifts than children (children: *M* = 1.72, *SE* = 0.31; adults: *M* = 2.57, *SE* = 0.31, *p* < 0.001, see Figure 2).

Moreover, within-groups comparisons show that the visuo-tactile version of RHI, in the absence of movement (Version 3), induced a larger proprioceptive drift in adults, which was nearly to zero in the other two action-based versions (*p* < 0.001). In children, the larger effect emerged in the two action-based versions of the RHI, namely when children actively stroked the rubber hand with (Version 1, *p* < 0.04) or without visual feedback (*p* = 0.001), as compared to the purely sensory version (Version 3); on the contrary, no difference emerged between Version 1 and 2 (*p* = 0.08).

## Discussion

In this study, we explored the contribution of the motor system, in particular of self-generated actions, in shaping and maintaining a coherent sense of self across development by using a novel, motor-based version of the RHI. We showed that action may either promote or disrupt the sense of body ownership depending on age, differently affecting the explicit and implicit self-location component.

In children, the subjective feeling of ownership over the rubber hand is overall similar in the three RHI versions, hence actively stroking one’s own hand does not significantly affect the illusory, subjective, experience of embodiment of the rubber hand. Instead, when children act on the rubber hand (i.e., being the agent of the delivered touch), the active movement promotes an efficient binding of the visual information (i.e., the seen rubber hand) and the tactile and proprioceptive inputs (i.e., the participant’s hand), necessary to recalibrate self-location following the embodiment of the rubber hand. Such effect is not dependent upon visual capture of attention by the participants’ own hand moving. Indeed, in the absence of active movements (Version 2), children lack to integrate multisensory signals, and thus the illusion does not shift their self-location. Therefore, at least until the preschool years, active movements selectively modulate the proprioceptive drift, but not the explicit feelings of owning the rubber hand, as assessed through the questionnaire.

The stability of self-reports in children is in line with previous studies in children of similar age (Cowie et al., 2013; Nava et al., 2017), corroborating the notion that the abstract representation of the body (also termed “Body Image”), which distinguishes between objects that may or may not be part of one’s body (Tsakiris, 2010), is likely innate, and shapes the conscious perception of feeling the rubber hand as one owns hand.

In adults, we found an opposite pattern, with action dramatically disrupting both the subjective sense of body ownership, and its implicit self-location component. Our findings complement previous evidence from the adult literature documenting the link between the sense of body ownership and the motor system activity. In clinical populations, it is the frequent association between movement disorders and a more malleable sense of body ownership: brain-damaged patients with hemiplegia (Burin et al., 2015), multiple sclerosis (Nava et al., 2017) or spinal cord injury (Scandola et al., 2014) all are more prone to the illusory effects the RHI. It is also noteworthy that somatoparaphrenia, a delusion of disownership of controlesional body parts, has been reported with a few exceptions, in right-brain-damaged patients, with motor deficits (Vallar and Ronchi, 2009; Bolognini et al., 2014). In the same vein, in healthy adults, reducing temporarily the level of activation of the motor cortex (with TMS or limb immobilization) attenuates the sense of body ownership, in turn making subjects more prone to incorporate an alien body part (Fossataro et al., 2018). Overall, this evidence is specular to the present one: here we showed that the activation of the motor system (through action) disrupts the RHI in adults, while in the above mentioned studies the opposite occurs, with a reduced (or even abolished in the case of permanent injuries) motor system activation increasing RHI effects. One possible explanation is that in adulthood, the presence of movement-related signals are able to lessens the impact of conflicting multisensory signals shaping the sense of body ownership.

Our results could also be interpreted in terms of attention to either visuo-motor or proprioceptive cues. Indeed, the participants were asked to stroke the visible fake hand (i.e., visuo-motor feedback), while passively feeling the stroke on the real hand (i.e., proprioceptive feedback), which may have automatically shifted their attention to the visuo-motor component. If this were the case, it suggests that visuo-motor integration may be particularly strong in children; strong enough to abolish proprioceptive cues, so that the task would be made solely following the former cues. In other words, instead of promoting the binding of visuo-proprioceptive signals, attention to the rubber hand may have added salience to the visuo-motor cues.

In adults, the same attentional mechanism may have favored proprioceptive cues because the active stroking of the fake hand may have heighten the awareness of ‘fakeness’ of the rubber hand. This, in turn, may have strengthens awareness over the real hand and thus abolished any recalibration of owns hand felt position.

An alternative interpretation of our results regards the possibility that sensory attenuation of self-produced tactile stimulations may strongly change throughout development. Studies in animal models have reported weaker neuronal responses to self-produced in comparison to externally generated stimuli across sensory modalities (Curio et al., 2000; Cullen and Roy, 2004), which is particularly striking in the case of self-tickling in human adults. Indeed, most adult individuals are insensitive to self-tickling, while many are when the tickling is done by someone else. From a neuronal point of view, this has been shown to correspond to weaker activity in the somatosensory cortices when the tickling or simply the touch is self-generated vs. externally produced (Blakemore et al., 1998, 2000; Hesse et al., 2010). Computational models suggest that this attenuation may be due to sensory predictions made by an internal forward model of the motor system. In other words, when the brain programs a movement, it also immediately predicts the sensory consequences of it. If the predicted and actual sensory feedbacks perfectly match, then the brain will alter the sensory signals online, and code the actions as self-produced. On the contrary, if there is a mismatch between predicted and actual sensory feedback, the brain will code the actions as non-intended, thus likely coming from an external source (from here the sensation of being tickled, for example).

Applied to our data, such evidence suggests that adults do not perceive the illusion because the self-produced strokes on the rubber hand increase self-awareness. In other words, adults expect that their own movements would cause a tactile sensation in correspondence to their own hand. Because their real hand is spatially misaligned with respect to the seen rubber hand, this causes a mismatch between predicted and actual sensory feedback, thus disrupting embodiment of the rubber hand.

In this view, the opposite pattern observed in children suggests a lower sensory attenuation in children, at least until 5 years of age, which may impair the capacity of dissociating sensory signals resulting from own vs. externally generated actions. Children’s inability to predict the consequences of their actions strengthen the RHI, as measured through the proprioceptive drift. While it is difficult to conclusively state whether larger drifts are really caused by predictive (or postdictive) mechanisms, and how these models causally interacts with self-location and body ownership, future studies should investigate how voluntary motor control generates sensory expectations in early development, how these expectations are compared with actual sensory feedback and whether they allow children to learn and distinguish between internal and external (bodily) events.

## Conclusion

In conclusion, the activation of a motor representation of one’s own body through action may recalibrate coherence among afferent sensory signals, in turn shaping the sense of body ownership in childhood and adults. At least until the preschool years, the immature sense of body ownership is strongly action-based, hence actions facilitate crossmodal interactions based on which a coherent sense of body ownership can emerge. Once the sensorimotor system has reached its maturity, the motor representation dominates bodily self-consciousness, lowering the susceptibility to conflicting sensory information that may cause body disembodiment. The sensory-based body representation dynamically interfaces with the motor system across the life span, supporting the view that self- representation and body awareness are not purely sensory or motor, but rather sensory and motor (Rizzolatti et al., 2002; Serino et al., 2013).

## Author Contributions

EN and NB conceived and designed the experiments. CG and AB carried out the experiments. EN analyzed the data and drafted the manuscript, revised by NB. All authors read and approved the final version of the manuscript.

## Conflict of Interest Statement

The authors declare that the research was conducted in the absence of any commercial or financial relationships that could be construed as a potential conflict of interest.
